# Supplementary material for: Revised International Staging System (R-ISS) stage-dependent analysis uncovers oncogenes and potential immunotherapeutic targets in multiple myeloma (MM)
Source: eLife. 2022 Oct 31;11:e75340. doi: 10.7554/eLife.75340 (PMC9674343; doi:10.7554/eLife.75340)
Supplement: Supplementary file 1. [file elife-75340-supp1.zip › Supplementary file 1.doc]

```
gene    Cluster_6_in_MM_III_mean        Cluster_6_in_MM_I_mean  log2FC  p_val   p_val_adj       pct.1   pct.2
RAB13   0.007780544     0.545459842     -0.616854657    0       0       0.003   0.584
IGHD    9.62E-05        1.934449815     -1.552951277    0       0       0       0.74
MC4R    0       0.569359114     -0.65017552     0       0       0       0.351
IGLC3   0.042182577     189.7444472     -7.515889208    0       0       0.018   0.935
AC004556.1      0.002036628     0.538573636     -0.618658246    0       0       0.001   0.571
FRZB    0.00815485      1.779142104     -1.462922355    0       0       0.005   0.727
IGLV5-45        0.000616686     6.047256357     -2.816172281    0       0       0       0.753
BCHE    0       1.208830779     -1.143282896    0       0       0       0.649
LINC01287       0.00593611      0.77464051      -0.818988129    0       0       0.006   0.61
DEFA3   0.062244128     3.448140698     -2.066087053    3.46E-261       9.76E-257       0.008   0.481
IGLC2   1.955582438     763.4758991     -8.014884748    8.99E-242       2.53E-237       0.062   1
CAMP    0.013589067     3.142537748     -2.031041975    2.45E-231       6.91E-227       0.002   0.273
SEL1L3  0.041002677     0.982482458     -0.929334322    6.24E-214       1.76E-209       0.044   0.844
RPS4Y1  0.000781494     0.749909071     -0.806152942    1.96E-211       5.51E-207       0.001   0.195
DPEP1   0.028332553     0.957795342     -0.928923067    9.77E-205       2.75E-200       0.027   0.662
MNDA    0.011537872     0.617781639     -0.677466557    1.36E-196       3.84E-192       0.005   0.338
LCN2    0.010245109     0.772127187     -0.810776784    4.21E-195       1.19E-190       0.002   0.247
S100A8  0.253932098     21.56931717     -4.169831639    4.58E-186       1.29E-181       0.044   0.779
IGLV1-40        9.00E-05        1.390741212     -1.257328079    3.45E-171       9.71E-167       0       0.143
S100A9  0.21601344      10.35896368     -3.223600139    1.64E-150       4.62E-146       0.054   0.766
RETN    0.013371402     1.009217658     -0.987470839    3.13E-144       8.82E-140       0.004   0.26
S100A12 0.027856842     1.211628194     -1.105469526    2.61E-141       7.34E-137       0.009   0.338
LTF     0.014095002     1.053462412     -1.017865727    1.32E-132       3.73E-128       0.005   0.26
ANKRD37 0.038185806     0.921784016     -0.888381536    2.76E-116       7.77E-112       0.042   0.597
ICAM3   0.086725367     1.31544242      -1.091300487    3.29E-115       9.28E-111       0.064   0.727
LYZ     0.058920686     1.294474251     -1.115569083    3.88E-113       1.09E-108       0.02    0.429
AHSP    0.016986        0.939310743     -0.931244172    1.77E-105       4.99E-101       0.001   0.13
HBA2    0.089700796     0.885722577     -0.791185384    2.81E-99        7.92E-95        0.014   0.338
PPDPF   0.095837671     0.945636566     -0.828208144    5.20E-94        1.47E-89        0.084   0.753
HBA1    0.074814358     1.539372701     -1.240384655    2.91E-93        8.19E-89        0.013   0.312
ATF5    0.09851889      2.042078058     -1.469497494    1.32E-88        3.71E-84        0.092   0.74
FAM107B 0.066942759     0.730477668     -0.697687546    6.32E-79        1.78E-74        0.053   0.558
CAV1    0.138349117     1.430975988     -1.094592562    3.14E-74        8.84E-70        0.127   0.792
PAGE5   0.097427352     1.194803594     -0.999966405    3.28E-65        9.24E-61        0.045   0.468
ARHGDIB 0.073053657     0.707697192     -0.670329961    1.22E-61        3.45E-57        0.044   0.455
ARHGAP18        0.136527171     1.235116536     -0.975717879    1.49E-61        4.19E-57        0.129   0.74
MIB2    0.15023411      0.932200959     -0.748317623    4.95E-57        1.39E-52        0.132   0.74
IFITM1  0.098405514     0.785186401     -0.700663948    3.72E-56        1.05E-51        0.059   0.506
PHGDH   0.293589927     2.540256079     -1.452473369    1.88E-47        5.28E-43        0.213   0.792
DEK     0.182316793     1.487300404     -1.072964113    5.45E-47        1.53E-42        0.135   0.675
CD74    0.034042765     0.717399206     -0.731929577    1.18E-45        3.34E-41        0.02    0.26
RPL14   12.08605754     34.2059092      -1.427787082    5.47E-45        1.54E-40        0.972   1
EBPL    0.216702025     0.957456606     -0.686004436    1.63E-44        4.59E-40        0.199   0.831
CD48    16.60488014     2.006247867     2.549939535     1.69E-44        4.77E-40        0.973   0.857
CCND2   0.80745421      10.47914071     -2.666983648    2.57E-44        7.25E-40        0.386   0.922
ZFP36L2 0.490807468     5.739225419     -2.176488832    7.17E-43        2.02E-38        0.319   0.909
IGKC    1047.36883      138.0002258     2.914987234     1.60E-42        4.51E-38        1       0.844
RPL32   22.08609043     54.83019667     -1.274021761    3.25E-42        9.15E-38        0.996   1
RPL26   15.97100214     48.18567787     -1.535166527    4.27E-42        1.20E-37        0.984   1
LRRC75A 0.144703249     1.718520028     -1.247847812    9.66E-42        2.72E-37        0.101   0.558
HLA-A   29.99076561     7.452935968     1.87431397      8.89E-41        2.50E-36        0.994   0.974
DPP7    0.309463821     1.388842091     -0.867335291    9.49E-41        2.67E-36        0.238   0.857
CTAG2   5.6050141       0.086454621     2.603933724     5.08E-40        1.43E-35        0.888   0.052
ITM2A   0.652065568     3.911754487     -1.571967503    5.70E-40        1.60E-35        0.276   0.87
TSPO    0.225860804     1.17373558      -0.826381287    3.63E-39        1.02E-34        0.17    0.727
RPS4X   22.82878602     53.45563324     -1.192375844    4.08E-38        1.15E-33        0.993   1
C12orf45        0.502288962     2.199032462     -1.090473293    5.99E-38        1.69E-33        0.356   0.922
RPL15   21.08890749     48.56306078     -1.165943219    9.03E-38        2.54E-33        0.992   1
FXYD5   0.52018577      2.597774462     -1.242857115    1.43E-37        4.02E-33        0.331   0.896
RPL22L1 0.704446083     4.342315489     -1.648162214    1.96E-37        5.51E-33        0.45    0.935
RPL6    15.62528101     36.71389279     -1.181717345    2.58E-37        7.28E-33        0.985   1
MYO6    0.180808568     1.044454405     -0.791940794    5.95E-37        1.68E-32        0.168   0.688
LINC01480       9.884579607     0.869029945     2.541924099     7.89E-37        2.22E-32        0.927   0.584
RPS2    33.94284197     101.9209111     -1.558467278    1.33E-35        3.74E-31        0.997   1
HLA-B   41.30165634     10.80662484     1.841117553     4.06E-35        1.14E-30        0.995   0.974
SNHG25  4.649486723     0.157346773     2.287298599     4.23E-35        1.19E-30        0.858   0.182
IGKV2D-28       3.507532939     0       2.172338034     5.95E-34        1.68E-29        0.801   0
RPL34   19.28085856     39.02217039     -0.980680679    1.42E-33        4.01E-29        0.993   1
PTP4A3  5.919293039     0.399717682     2.305488772     3.84E-33        1.08E-28        0.868   0.416
RPSA    11.5687838      25.68506059     -1.08618723     4.96E-33        1.40E-28        0.963   1
UQCRB   11.00636581     4.458109683     1.137326238     5.47E-33        1.54E-28        0.97    0.961
RPL21   16.90095804     34.33820056     -0.981191778    1.94E-32        5.46E-28        0.987   1
RPS12   33.30313905     73.57013342     -1.120257321    2.08E-32        5.86E-28        0.999   1
EEF1A1  40.9523159      109.5195137     -1.39747876     2.20E-32        6.18E-28        1       1
JCHAIN  146.3627584     57.74200773     1.326907491     3.98E-32        1.12E-27        1       1
COX6C   9.627015472     3.330114865     1.295259281     1.16E-31        3.28E-27        0.956   0.974
NME4    0.318150303     1.195863877     -0.73627374     8.20E-31        2.31E-26        0.251   0.779
IKBIP   0.227751559     0.970352668     -0.682435222    1.21E-30        3.40E-26        0.215   0.74
RPL5    17.88102613     44.8321613      -1.279423147    1.80E-30        5.06E-26        0.99    1
CTSS    0.406554705     1.400246283     -0.77101678     3.05E-30        8.59E-26        0.249   0.805
RPL19   30.19040868     58.02582738     -0.920243903    6.89E-30        1.94E-25        0.998   1
RPL12   22.09695419     61.6877467      -1.440480858    1.05E-29        2.97E-25        0.994   1
WARS    7.764080308     0.651224362     2.408066543     2.80E-29        7.89E-25        0.867   0.494
IGHG2   1.95137278      46.38289582     -4.004908312    7.10E-29        2.00E-24        0.5     0.831
HBD     7.665298845     0.896645391     2.191799537     8.73E-29        2.46E-24        0.852   0.156
SPAG4   0.257442518     1.099729279     -0.73971088     1.69E-28        4.77E-24        0.214   0.688
MZB1    12.96893863     37.43000898     -1.460010906    3.61E-27        1.02E-22        0.955   0.961
PIM2    3.297825671     0.229106235     1.805997348     7.53E-27        2.12E-22        0.781   0.195
ATP5IF1 10.66289645     4.577633998     1.064200949     1.03E-26        2.89E-22        0.967   0.974
RPS3A   33.76494952     63.89286571     -0.900426383    1.37E-26        3.85E-22        0.999   1
RPL18   30.37302208     54.55008451     -0.8242646      2.77E-26        7.82E-22        0.997   1
HLA-C   26.25137534     11.77015435     1.093553079     2.98E-26        8.40E-22        0.989   0.987
CADM1   4.450756216     0.323422517     2.042182666     5.04E-26        1.42E-21        0.793   0.286
RNASET2 0.712512866     2.133954285     -0.871869308    7.45E-26        2.10E-21        0.442   0.909
LETM1   4.445608793     0.218797948     2.159634369     8.67E-26        2.44E-21        0.773   0.273
RPL9    20.91534195     35.98085448     -0.75483737     1.22E-25        3.45E-21        0.995   1
SPCS1   5.028259456     12.43268653     -1.155934455    1.79E-25        5.04E-21        0.835   0.987
ENO1    8.408177291     2.791159796     1.31127598      2.51E-25        7.06E-21        0.938   0.974
ITM2B   1.533270888     5.226915421     -1.297516328    3.01E-25        8.47E-21        0.636   0.948
COMMD6  0.59128099      1.679860375     -0.751969226    4.38E-25        1.23E-20        0.386   0.896
ANXA11  0.398233473     1.190513198     -0.64766363     4.40E-25        1.24E-20        0.303   0.805
RPL7A   39.41369233     67.70656876     -0.765603869    1.49E-24        4.21E-20        0.999   1
TMSB4X  36.77745585     13.46203069     1.385255403     2.06E-24        5.81E-20        0.994   0.974
TMSB10  17.6572022      8.821542723     0.925711099     3.02E-24        8.50E-20        0.983   0.987
DNAJC15 0.51068138      1.502360008     -0.72808995     3.32E-24        9.36E-20        0.353   0.844
RPS7    25.19779356     39.25693267     -0.619791942    5.46E-24        1.54E-19        0.995   1
FKBP11  2.949700412     9.939287342     -1.469703622    6.19E-24        1.74E-19        0.777   0.948
RPL29   23.12871613     46.77604797     -0.985536366    7.31E-24        2.06E-19        0.992   1
PMVK    3.194202052     0.490394239     1.492702361     9.52E-24        2.68E-19        0.813   0.532
CTHRC1  0.95912012      6.55914127      -1.948016495    1.19E-23        3.35E-19        0.466   0.753
UCHL1   1.950088603     0.018655649     1.534091846     1.35E-23        3.79E-19        0.661   0.026
TMEM238 0.668450149     2.147178448     -0.915550402    2.56E-23        7.22E-19        0.428   0.857
IGLL5   2.857040513     0.353776972     1.510504218     5.72E-23        1.61E-18        0.784   0.429
IDH2    4.823232568     0.970847726     1.563003925     7.89E-23        2.22E-18        0.86    0.74
TM9SF2  0.41420394      1.169642073     -0.617466876    1.53E-22        4.32E-18        0.322   0.766
PTMA    29.3171059      47.34659098     -0.673282127    2.10E-22        5.91E-18        0.997   1
KRTCAP2 3.546783352     9.876656767     -1.258317005    2.44E-22        6.87E-18        0.797   1
NES     4.103321697     0.012258838     2.333858348     3.49E-22        9.84E-18        0.631   0.013
RPL41   33.01828639     51.52941218     -0.626814971    4.44E-22        1.25E-17        0.999   1
XBP1    18.08969757     7.852267132     1.108675354     5.49E-22        1.55E-17        0.971   0.961
RPS13   20.7968874      35.07030872     -0.726689638    5.53E-22        1.56E-17        0.994   1
RPS9    20.33829238     33.77591191     -0.704643619    1.02E-21        2.87E-17        0.992   1
PLPP5   0.893021128     2.414018421     -0.85078033     1.71E-21        4.81E-17        0.497   0.909
DNAJC1  2.000622003     4.653908144     -0.913986855    1.80E-21        5.06E-17        0.681   0.974
PSMD4   3.886044887     1.062013302     1.244613478     1.80E-21        5.06E-17        0.851   0.844
FABP5   5.124828498     1.101513357     1.543240822     1.98E-21        5.57E-17        0.856   0.688
SLC44A1 0.763007652     2.379242684     -0.938661226    2.09E-21        5.87E-17        0.468   0.896
ATP5MC2 4.101868709     8.071356749     -0.830292569    4.02E-21        1.13E-16        0.86    1
RPL28   42.22774532     65.43119678     -0.619903315    4.78E-21        1.35E-16        0.999   1
TSTD1   3.862465401     0.962008251     1.309356876     8.34E-21        2.35E-16        0.842   0.636
POU2AF1 2.842021944     0.442265152     1.41352934      1.51E-20        4.25E-16        0.772   0.481
YBX1    20.16527903     11.4412722      0.766565491     3.20E-20        9.02E-16        0.989   0.987
HLA-F   2.421832917     0.273076409     1.426450306     3.34E-20        9.42E-16        0.719   0.221
RPS27   67.77222233     44.68076835     0.590239049     4.30E-20        1.21E-15        1       1
RRBP1   2.375725766     6.605027544     -1.171755765    4.34E-20        1.22E-15        0.699   0.974
HMGA1   5.217273984     1.574485602     1.271997955     5.86E-20        1.65E-15        0.876   0.857
RPL13A  23.62391261     40.8337488      -0.76460727     1.18E-19        3.31E-15        0.994   1
TPT1    30.64991334     59.82988685     -0.942578771    1.29E-19        3.62E-15        0.999   1
TNFRSF13B       2.317758199     0.094173173     1.600367656     1.33E-19        3.75E-15        0.634   0.091
NENF    0.649800859     1.560923709     -0.634372382    1.53E-19        4.31E-15        0.417   0.883
HLA-E   7.267771286     2.815805642     1.11551079      1.69E-19        4.75E-15        0.907   0.857
IFI6    0.381311844     1.899300573     -1.069665848    1.74E-19        4.90E-15        0.263   0.675
EEF1B2  17.54783519     27.32842643     -0.610999654    2.09E-19        5.88E-15        0.988   1
PCBP2   1.167379846     2.880939867     -0.840454063    2.29E-19        6.45E-15        0.58    0.961
MT-ND3  39.37294689     18.37052113     1.059526128     3.13E-19        8.82E-15        0.995   0.974
RTRAF   1.003326119     2.338571345     -0.736833576    3.73E-19        1.05E-14        0.546   0.883
PRRC2C  4.845806201     1.474968565     1.2399918       5.93E-19        1.67E-14        0.823   0.831
ASS1    0.822070457     3.310494093     -1.242274501    6.52E-19        1.84E-14        0.386   0.74
RPL24   21.07350307     32.60955755     -0.606555947    6.97E-19        1.96E-14        0.993   1
LILRB4  1.527516508     0.034572035     1.288686414     9.44E-19        2.66E-14        0.588   0.039
RPL31   5.767514558     9.993533843     -0.69995722     1.15E-18        3.23E-14        0.91    0.987
RACK1   15.78860061     27.27436507     -0.752012646    1.16E-18        3.27E-14        0.983   1
ATP5MG  10.25094027     5.667118601     0.75491028      1.29E-18        3.62E-14        0.966   0.987
UFC1    4.641338435     1.831989254     0.994221698     1.41E-18        3.98E-14        0.879   0.922
YBX3    1.904571142     0.266201397     1.197818274     1.69E-18        4.76E-14        0.694   0.156
RAB11FIP2       2.859134077     0.240045036     1.63788465      2.09E-18        5.90E-14        0.699   0.364
FOSB    0.283529164     1.858838699     -1.155313146    3.32E-18        9.35E-14        0.173   0.506
PEBP1   2.102749953     4.203913905     -0.746049658    4.88E-18        1.38E-13        0.706   0.948
EIF4B   2.216387319     5.122927832     -0.928780532    6.32E-18        1.78E-13        0.731   0.948
RPS5    24.91047034     47.70142102     -0.910428664    6.61E-18        1.86E-13        0.995   1
AC012236.1      2.354360199     0.323658814     1.341506316     7.69E-18        2.17E-13        0.724   0.39
IMPDH2  0.758592502     1.947393562     -0.745018492    7.86E-18        2.21E-13        0.462   0.844
NSD2    3.355776334     0.570184526     1.471995762     1.23E-17        3.47E-13        0.744   0.584
AIG1    0.610768181     1.524443352     -0.648216426    1.24E-17        3.50E-13        0.417   0.818
MYC     0.499753887     1.957148135     -0.979480745    1.37E-17        3.86E-13        0.306   0.714
LDHA    6.541145104     2.6360636       1.052406175     1.55E-17        4.37E-13        0.91    0.935
TRAM1   6.737936155     2.890516133     0.991987263     1.97E-17        5.54E-13        0.875   0.974
CYTOR   4.074534378     1.139300436     1.246136348     2.66E-17        7.50E-13        0.816   0.753
RAB4A   0.648358432     1.493456475     -0.597117032    4.22E-17        1.19E-12        0.42    0.805
TMEM59  1.903219774     3.796072067     -0.724199549    8.40E-17        2.37E-12        0.679   0.935
LAX1    1.749952807     0.166359691     1.237394094     8.83E-17        2.49E-12        0.639   0.208
LAMP5   0.049960955     0.732248543     -0.722310264    1.00E-16        2.82E-12        0.042   0.234
TMED2   1.120370643     2.358607091     -0.663546559    1.08E-16        3.04E-12        0.571   0.948
NDUFB1  3.838507376     1.35328084      1.039888559     1.13E-16        3.19E-12        0.854   0.831
LGALS1  8.747011962     2.949704778     1.303215192     1.16E-16        3.28E-12        0.91    0.883
CALR    8.823187574     4.498499193     0.837153357     1.23E-16        3.47E-12        0.914   0.974
SELENOF 0.618336856     1.46681986      -0.608140431    1.27E-16        3.58E-12        0.416   0.831
MAPKAPK2        2.790146182     0.574235607     1.267602016     1.35E-16        3.79E-12        0.758   0.597
SLC25A5 9.701997842     5.16279931      0.796222427     1.39E-16        3.92E-12        0.947   1
RPL17   2.617915647     4.874971534     -0.69942309     1.42E-16        4.00E-12        0.783   0.974
MLEC    1.310971827     2.65974242      -0.66324244     1.44E-16        4.04E-12        0.615   0.948
HSPB1   0.862327599     1.826987117     -0.602158431    2.21E-16        6.22E-12        0.406   0.831
PSMD7   4.279174969     1.703972269     0.965232126     2.54E-16        7.15E-12        0.854   0.922
ZBTB38  4.019507297     1.214903399     1.180301981     3.00E-16        8.44E-12        0.804   0.818
C1QBP   1.512035002     3.104384848     -0.708309445    3.98E-16        1.12E-11        0.627   0.948
GOLM1   1.334591691     0.012330393     1.205490034     5.08E-16        1.43E-11        0.519   0.026
PARK7   6.975379033     3.658079554     0.775817806     6.92E-16        1.95E-11        0.926   0.961
JSRP1   0.851092795     2.501534278     -0.919609991    7.54E-16        2.12E-11        0.454   0.74
LY6E    6.845057104     2.866365893     1.020805775     9.38E-16        2.64E-11        0.899   0.909
EVI2B   1.31356382      2.657694362     -0.660817632    1.14E-15        3.21E-11        0.46    0.909
TIMD4   1.143837189     0       1.100195346     1.38E-15        3.88E-11        0.492   0
MT-ND2  52.34541933     29.05344415     0.82783406      1.82E-15        5.13E-11        0.998   0.974
APRT    5.133045682     2.089033276     0.989448285     2.98E-15        8.41E-11        0.883   0.922
COX17   3.613747023     1.386446633     0.951074827     3.27E-15        9.22E-11        0.85    0.753
MAP1B   1.0316312       0       1.022638535     4.78E-15        1.35E-10        0.479   0
ENSA    2.857317111     0.934018146     0.995996424     6.22E-15        1.75E-10        0.791   0.649
HIPK2   2.545134188     0.592343558     1.154688597     7.81E-15        2.20E-10        0.713   0.403
MAF     0.855440235     0       0.891761531     1.14E-14        3.21E-10        0.471   0
HSPA5   13.15915801     6.441612453     0.928048312     1.29E-14        3.64E-10        0.93    0.961
PSMB2   3.879128218     1.325747061     1.068929193     1.43E-14        4.03E-10        0.832   0.87
YWHAZ   3.383837704     1.049439942     1.096964676     1.48E-14        4.17E-10        0.808   0.818
RBM8A   4.935901761     2.200087086     0.891356049     1.49E-14        4.19E-10        0.885   0.87
UAP1    5.034425892     1.56736773      1.23292657      1.80E-14        5.07E-10        0.834   0.857
ZNF593  2.636411415     0.724736448     1.076139507     2.14E-14        6.02E-10        0.772   0.714
SRGN    5.78706047      2.523386416     0.945824161     2.27E-14        6.39E-10        0.865   0.883
MCL1    3.754837009     1.099346738     1.179455419     2.81E-14        7.90E-10        0.778   0.74
HNRNPH1 0.966353666     2.27748887      -0.737068055    2.91E-14        8.19E-10        0.529   0.896
RPS11   20.789463       32.07000039     -0.601892421    3.87E-14        1.09E-09        0.993   1
TPD52   3.468697552     1.246157306     0.992395437     4.58E-14        1.29E-09        0.809   0.766
PRDX6   4.73418498      1.995000845     0.937032035     4.99E-14        1.41E-09        0.866   0.896
ISG15   1.224207343     2.882492784     -0.803691959    5.93E-14        1.67E-09        0.417   0.844
ANP32E  4.834571453     1.959283073     0.979378989     6.85E-14        1.93E-09        0.852   0.87
GUK1    4.855299856     2.476819797     0.75197476      1.05E-13        2.95E-09        0.872   0.961
NDUFS5  7.204032526     4.064865219     0.695809334     1.07E-13        3.01E-09        0.934   0.961
ISG20   5.42176673      2.214413078     0.998414921     1.12E-13        3.15E-09        0.864   0.792
TRAF3IP3        1.066717981     0.07704438      0.940263837     1.32E-13        3.72E-09        0.498   0.052
RPL3    39.22255802     64.09032864     -0.694438363    1.37E-13        3.87E-09        0.999   1
DDIT4   0.908966096     2.848923739     -1.011663606    1.38E-13        3.90E-09        0.434   0.727
GTF3A   1.700406187     3.160583946     -0.623609598    1.54E-13        4.33E-09        0.659   0.961
SON     1.483603264     2.795452135     -0.611837025    1.77E-13        4.99E-09        0.641   0.974
COX8A   5.641876021     3.076136925     0.704388278     1.85E-13        5.20E-09        0.9     0.961
CCDC34  0.701044025     1.581858924     -0.601989692    1.95E-13        5.49E-09        0.424   0.779
PSMA4   3.759453462     1.336352407     1.026538014     2.08E-13        5.85E-09        0.833   0.844
IER2    4.218653289     0.930530755     1.434680019     2.32E-13        6.52E-09        0.729   0.584
AURKAIP1        4.765390431     2.355362076     0.780949855     2.32E-13        6.54E-09        0.884   0.935
HIST1H2AJ       0.143405195     0.729787678     -0.597258216    3.37E-13        9.48E-09        0.13    0.403
BEX3    1.171203957     0.209830695     0.843690085     4.32E-13        1.22E-08        0.537   0.091
UBALD2  4.039508218     1.077224259     1.278625975     4.38E-13        1.23E-08        0.777   0.74
PDK1    0.828521255     1.93477711      -0.682573541    5.15E-13        1.45E-08        0.476   0.805
RHOB    1.677050375     0.104730182     1.276950237     6.05E-13        1.70E-08        0.524   0.104
NDUFA1  4.396241014     2.016249524     0.839199002     1.08E-12        3.05E-08        0.87    0.87
ATP5F1E 13.38251634     8.548250227     0.591007829     1.51E-12        4.26E-08        0.982   1
HINT1   13.62848822     8.281287225     0.65638387      1.60E-12        4.50E-08        0.977   0.987
MT-CO1  35.63751698     60.06766501     -0.737086952    1.78E-12        5.02E-08        0.993   0.974
CLIC1   6.17511087      3.127464067     0.797745469     1.83E-12        5.15E-08        0.901   0.948
CD180   0.956561496     0.022951918     0.93558212      2.75E-12        7.75E-08        0.459   0.065
ITGB7   1.21994802      0.07123113      1.051256105     2.78E-12        7.83E-08        0.487   0.078
ARPC2   2.953389645     4.987909154     -0.59896218     2.82E-12        7.94E-08        0.784   1
TPM3    4.594712972     2.532514918     0.663368465     2.92E-12        8.23E-08        0.878   0.948
PABPC1  13.07566125     8.071028944     0.633864587     3.38E-12        9.54E-08        0.971   0.961
FBP1    2.44117529      0.333484667     1.367700147     4.15E-12        1.17E-07        0.638   0.377
CDV3    4.962794659     2.51956781      0.760590373     4.43E-12        1.25E-07        0.867   0.922
HIST1H3G        0.286415633     2.293488997     -1.356259887    4.44E-12        1.25E-07        0.195   0.481
NDUFA13 4.381089735     2.062145245     0.81335565      7.71E-12        2.17E-07        0.871   0.935
TIMM8B  3.228545812     1.20969457      0.936314639     8.95E-12        2.52E-07        0.803   0.818
RPL36AL 4.284825594     7.048388527     -0.60684409     1.01E-11        2.83E-07        0.865   1
DPM3    2.938763795     1.036513711     0.951641375     1.27E-11        3.58E-07        0.79    0.831
ODC1    1.706300719     3.265112866     -0.656261762    1.28E-11        3.59E-07        0.665   0.948
PSMB9   3.474810942     1.349110246     0.929712308     1.57E-11        4.43E-07        0.813   0.766
CD44    1.767493518     0.477136976     0.905776323     1.64E-11        4.62E-07        0.644   0.234
EIF3L   1.824181211     3.426144705     -0.648217957    1.65E-11        4.65E-07        0.695   0.935
MT-ATP6 68.48531946     41.38266386     0.713233936     1.69E-11        4.75E-07        0.999   0.974
TAGLN2  2.479639285     0.910019738     0.865350211     1.72E-11        4.86E-07        0.736   0.649
HRASLS2 1.298243603     0.035890354     1.149660419     1.97E-11        5.54E-07        0.435   0.052
UBB     7.145520037     4.283109164     0.624619588     3.21E-11        9.05E-07        0.929   0.961
ATP6V0B 4.520901534     2.303676851     0.740831294     4.42E-11        1.24E-06        0.839   0.922
MSN     1.555381304     0.344615128     0.926345294     8.25E-11        2.32E-06        0.63    0.364
NDUFS2  2.111212522     0.661247719     0.905209727     8.92E-11        2.51E-06        0.73    0.675
MAN1A1  0.941662191     2.082990871     -0.667038394    1.03E-10        2.89E-06        0.505   0.792
PSAP    1.850964596     3.410312966     -0.629430913    1.23E-10        3.47E-06        0.689   0.935
MALAT1  285.6668658     143.1576031     0.991728232     1.55E-10        4.36E-06        1       0.974
CYCS    4.569809511     2.303440152     0.75365878      1.55E-10        4.37E-06        0.86    0.935
CCL3    1.405819389     0       1.26652834      1.71E-10        4.80E-06        0.363   0
CHCHD7  1.492790139     0.308650356     0.92968174      1.78E-10        5.00E-06        0.638   0.429
IL32    2.895997753     0.265128457     1.622708967     1.80E-10        5.08E-06        0.42    0.039
SHROOM3 0.524115493     0       0.607972231     1.90E-10        5.35E-06        0.361   0
PXDN    0.519392528     0.004229195     0.597406058     2.04E-10        5.75E-06        0.372   0.013
GRK6    1.72429637      0.426333312     0.933572502     2.59E-10        7.29E-06        0.668   0.545
TCF4    1.503370851     0.352159617     0.888606562     2.59E-10        7.29E-06        0.614   0.351
CPNE5   0.911855165     0.124714692     0.765414157     2.60E-10        7.33E-06        0.495   0.143
GPRIN3  1.440510267     0.282694043     0.928005731     2.68E-10        7.55E-06        0.594   0.312
TBCA    3.593103023     1.714865928     0.75858819      3.01E-10        8.47E-06        0.842   0.87
HIST1H1B        0.795367003     3.893757819     -1.446663923    3.14E-10        8.85E-06        0.354   0.623
C11orf96        0.755243625     0       0.811671288     3.34E-10        9.40E-06        0.354   0
HSP90AA1        16.61103702     9.518169467     0.743596218     3.48E-10        9.80E-06        0.972   0.987
EIF2S2  4.73664989      2.646007409     0.653890979     3.91E-10        1.10E-05        0.882   0.961
SLAMF7  3.196929906     1.148910552     0.965728937     5.00E-10        1.41E-05        0.756   0.831
MIF     4.525491657     7.538694222     -0.627912622    5.73E-10        1.61E-05        0.882   0.948
CCT3    5.940925481     3.389042705     0.661221733     5.77E-10        1.63E-05        0.902   0.961
HAX1    4.115389067     2.197547996     0.677877958     6.97E-10        1.96E-05        0.843   0.909
UQCR10  4.647660735     2.495231737     0.692265315     7.05E-10        1.99E-05        0.884   0.948
CHD7    0.968040305     0.144016116     0.782652391     7.48E-10        2.11E-05        0.526   0.234
SCAND1  2.662552898     1.063368812     0.827847881     7.50E-10        2.11E-05        0.779   0.766
FAM27C  0.94795731      0.114040719     0.806160096     9.07E-10        2.56E-05        0.496   0.182
PEG10   0.586801369     0       0.666121547     1.02E-09        2.88E-05        0.34    0
TMED9   3.218816132     1.560778272     0.720255871     1.07E-09        3.00E-05        0.794   0.805
UQCR11  4.198551556     2.283740853     0.662769432     1.40E-09        3.96E-05        0.855   0.909
MT-ND4L 6.933183003     3.899076873     0.6953899       1.54E-09        4.34E-05        0.893   0.922
PSMB8   3.03359653      1.448866146     0.719952863     1.67E-09        4.71E-05        0.799   0.792
DERL1   2.757256428     1.13854953      0.81304696      2.01E-09        5.65E-05        0.765   0.792
TXNDC5  1.628806624     0.443309855     0.865026964     2.08E-09        5.86E-05        0.661   0.506
MINDY3  1.016350412     0.094772413     0.881115393     2.37E-09        6.68E-05        0.478   0.182
TAP1    2.049263851     0.641263211     0.893654367     2.57E-09        7.23E-05        0.694   0.571
MTDH    14.31441264     9.049843881     0.607716945     2.89E-09        8.14E-05        0.944   0.987
AL133467.1      0.651093097     0.061199265     0.637725887     2.90E-09        8.18E-05        0.394   0.052
CDK6    1.911632741     0.535319676     0.923289315     3.03E-09        8.53E-05        0.659   0.545
MYL12A  3.751785522     1.683577364     0.82431224      3.33E-09        9.37E-05        0.826   0.883
SSU72   2.018105093     0.716892871     0.813843021     3.48E-09        9.81E-05        0.724   0.688
RAD21   2.931842511     1.053767634     0.936932574     4.15E-09        0.000116909     0.747   0.818
CD320   2.759671538     0.973101472     0.930141475     4.42E-09        0.000124617     0.747   0.766
HSP90AB1        16.30948299     10.17790116     0.630913311     4.92E-09        0.000138515     0.975   1
CLEC7A  0.574425145     0.012304134     0.637182373     5.03E-09        0.000141715     0.337   0.013
RALGPS2 2.105446618     0.592380889     0.963615306     5.29E-09        0.000148979     0.677   0.545
IDH3A   1.01226529      0.161346463     0.793022084     5.43E-09        0.000153022     0.529   0.273
EIF3H   4.2183253       2.220575409     0.696268409     6.18E-09        0.000173984     0.869   0.948
CNDP2   1.517248632     0.362886124     0.885182697     6.49E-09        0.000182754     0.634   0.481
LAMC1   0.821654        0.139663277     0.676641332     6.84E-09        0.0001928       0.493   0.195
DEPP1   0.513599416     0.008580498     0.585657202     6.90E-09        0.000194446     0.331   0.013
MGMT    0.863421371     0.181253521     0.657635315     1.07E-08        0.000302332     0.495   0.169
MYBL2   1.262635778     0.300432961     0.79901234      1.33E-08        0.00037381      0.579   0.338
HOMER3  2.244759447     0.453805142     1.158277613     1.62E-08        0.000457536     0.599   0.416
DUSP23  0.719593633     0.094477622     0.651825216     1.64E-08        0.000460612     0.445   0.143
RCSD1   0.893811535     0.231102198     0.621342236     1.92E-08        0.000541033     0.485   0.156
SOX4    0.964272215     0.306607491     0.588169061     2.27E-08        0.000640391     0.479   0.143
MINOS1  2.922932559     1.342926197     0.743621024     2.60E-08        0.000731111     0.792   0.831
EPB41L2 0.550836227     0.026173488     0.595771684     2.90E-08        0.000817506     0.358   0.065
SLC39A4 0.637878671     0.062074044     0.624944141     2.97E-08        0.000838002     0.4     0.104
FAM50A  1.487986387     0.49230026      0.737440748     3.17E-08        0.000891674     0.643   0.468
JUN     4.092418646     1.192109601     1.216031096     3.18E-08        0.000897185     0.673   0.545
GHITM   2.637010604     1.12137001      0.777756854     3.60E-08        0.001013318     0.785   0.779
AIM2    0.8499833       0.154814478     0.679851147     5.22E-08        0.001469528     0.447   0.143
CKS1B   3.012010715     1.154266895     0.897128461     5.59E-08        0.001574218     0.754   0.74
IGHG4   0.296349872     3.352577251     -1.747414763    5.90E-08        0.001662622     0.128   0.338
PFDN2   2.847490535     1.315731176     0.732449991     6.47E-08        0.001823587     0.785   0.844
CSKMT   0.646833991     0.058038311     0.638303264     7.80E-08        0.00219707      0.394   0.117
MORF4L2 2.163186079     0.789901956     0.821497863     7.94E-08        0.002238082     0.721   0.675
ATP5MPL 4.100717224     2.240378175     0.654537927     1.01E-07        0.002856567     0.859   0.935
TP53INP1        1.155094879     0.354050642     0.670469689     1.14E-07        0.003225241     0.523   0.247
PLA2G16 1.793938926     0.485261345     0.911583676     1.25E-07        0.003521913     0.637   0.545
SEM1    2.768794109     1.325997248     0.696253592     1.33E-07        0.003736176     0.785   0.805
OXR1    0.853041348     0.160985421     0.674545218     1.48E-07        0.00417622      0.504   0.299
VDAC3   1.988804537     0.666043622     0.843142375     1.51E-07        0.004265777     0.689   0.61
ATP6V1F 2.227967254     0.946328855     0.729870453     1.58E-07        0.004462191     0.743   0.766
PAK1    0.687704602     0.093670718     0.625883975     1.94E-07        0.005452973     0.429   0.169
BMP8B   0.721072564     0.09673957      0.65008694      2.44E-07        0.00685951      0.429   0.182
VPS28   2.182091308     0.92161549      0.727655547     2.86E-07        0.008055913     0.741   0.779
CD79B   1.180908087     0.435400129     0.603476014     2.95E-07        0.008310614     0.558   0.312
ENY2    2.640215344     1.319496783     0.650211953     5.24E-07        0.014754991     0.781   0.805
DDX10   0.866525613     0.165249022     0.679717004     5.81E-07        0.016354765     0.49    0.286
PLIN2   0.710554304     0.124566068     0.605095483     5.87E-07        0.016549402     0.453   0.221
SPTBN1  2.030500968     0.581166975     0.938566576     5.96E-07        0.016781196     0.629   0.584
WIPI1   1.121464126     0.329176021     0.674528111     6.12E-07        0.017227321     0.559   0.351
NDUFV2  3.704694946     2.00459018      0.646932957     6.17E-07        0.017377589     0.83    0.909
ATP6AP1 1.005263721     0.293795553     0.632182325     6.87E-07        0.019353272     0.552   0.364
EMP3    4.146713209     1.831869467     0.861896626     8.10E-07        0.022828276     0.795   0.922
ETFB    2.722570379     1.275724454     0.709973239     9.48E-07        0.02669975      0.781   0.753
EIF3J   2.766237057     1.28754642      0.71932279      9.61E-07        0.027073554     0.783   0.831
RNF19A  1.733356952     0.574302957     0.795960677     1.15E-06        0.032465357     0.646   0.623
STAT1   2.053905914     0.650880474     0.887419945     1.37E-06        0.038541172     0.641   0.532
PSMC3   2.056304136     0.841237787     0.731112159     1.39E-06        0.039083438     0.722   0.675
MANF    3.099863646     1.69017889      0.607873818     1.67E-06        0.046950083     0.782   0.844
```
